# The effects and costs of the universal parent group program – all children in focus: a study protocol for a randomized wait-list controlled trial

**DOI:** 10.1186/1471-2458-13-688

**Published:** 2013-07-29

**Authors:** Lene Lindberg, Malin Ulfsdotter, Camilla Jalling, Eva Skärstrand, Maria Lalouni, Kajsa Lönn Rhodin, Anna Månsdotter, Pia Enebrink

**Affiliations:** 1 Department of public health sciences, Karolinska institutet, Stockholm, Sweden; 2 Department of clinical neuroscience, Karolinska institutet, Stockholm, Sweden; 3 Family and social welfare, Stockholm, Sweden

**Keywords:** Parenting programs, Parental self-efficacy, Child health, Development, Health promotion, Universal

## Abstract

**Background:**

In recent decades, parents have been involved in programs that aim to improve parenting style and reduce child behavior problems. Research of preventive parenting programs has shown that these interventions generally have a positive influence on both parents and children. However, to our knowledge there is a gap in the scientific literature when it comes to randomized controlled trials of brief, manual-based structured programs which address general parenting among the population, and focus on promoting health. A four-session universal health promotion parent group program named All Children in Focus was developed. It aims at promoting parental competence and children’s positive development with the parent–child relationship as the target. There is currently no randomized controlled trial existing of the program.

**Methods/Design:**

A prospective multicenter randomized wait-list controlled trial is being conducted. Approximately 600 parents with children ranging in age from 3–12 years have been recruited in eleven municipalities and city districts in the County of Stockholm, Sweden. Parents are randomized at baseline to an intervention group, which receives the program directly, or to a waiting-list control group, which participates in the program six months later. Changes in parenting and child health and development are assessed with measures immediately post-intervention and six months after the baseline. Observations of a minor group of parents and children are conducted to explore possible relations between parental reports and observed behaviors, as well as changes in the interaction between parent and child. Further, data collected within the evaluation will also be applied to evaluate the possible cost-effectiveness of the program.

**Discussion:**

This paper describes a study protocol of a randomized controlled trial. Except for the quantitative outcome measures to evaluate the effectiveness of All Children in Focus, this protocol also describes health economic and qualitative analyses to deepen the knowledge of the program. We further discuss some issues regarding the implementation of the program in municipalities and city districts.

**Trial registration:**

Current Controlled Trials ISRCTN70202532

## Background

Employment of various methods for supporting parents has been stressed as important to promote children’s health and development as well as to prevent different health problems. The support to parents can be delivered as programs or policies [1]. A common strategy or approach that has been deployed in recent decades is to involve parents in programs that aim at improving parenting style. Most of the research covering parental support programs focuses on established parental or child risk factors that could contribute to avoiding health or developmental problems for children rather than promoting health and development [2]. To prevent such negative outcome and to counterbalance risk factors, many programs aim for strengthening or developing protective factors [1-3]. Preventive programs such as the Incredible Years [4], COPE [5] and Triple P [6] were developed in a non-European context in the 1980s and 1990s.

Research of preventive parenting programs has shown that these interventions generally have a positive influence; first on children, by treating a variety of child behavior problems [7-12], and second, on parents, by improving parental behavior and skills [7,11,12]. Nevertheless, most of these interventions seem to address children’s or adolescents’ ill-health and disruptive or problem behavior, and tutor parents in methods and techniques for preventing or dealing with their children’s problematic behaviors [13-15]. However, studies of support for parents through health promotions of children’s mental development and well-being seem, to our knowledge, to be limited in the scientific literature when it comes to general parenting among the population [16,17]. This may indicate a shortage of development and research on interventions influenced by a salutogenic perspective, and that address the universal level of parenting. Such an intervention could strive for empowering parents in their general comprehension of confidence and capacity, and to strengthen their self-efficacy.

Parental empowerment in terms of formulating their own goals for what aspects they want to strengthen in their relationships with their children has been suggested as a topic for universal parental programs [18]. Encouraging parents to decide for themselves about what to emphasize in the upbringing of their children could, besides as an expression of empowerment, also be viewed as a tool to strengthen parental competence and parental self-efficacy [19]. In evaluations of parenting interventions, it is also valuable to study whether there are families that benefit more or less from interventions, as well as if there are variables mediating the outcome. Earlier research has investigated effects of moderators and mediators on outcome, where different factors such as family demographics (e.g., socioeconomic status), participation (e.g., attendance level), child variables (e.g., age, gender), and parent variables (e.g., mental health, positive parenting), have been shown to affect the outcome [12,20]. Since mediating and moderating factors mainly seem to be evaluated concerning prevention programs, it is also vital to investigate the potential effects of these in a universal health promotion program. Moreover, there is a lack of solid health economic evaluations of parent interventions, especially of universal interventions aiming at promoting mental health [21,22]. Since societal resources are scarce, priorities must be made. By identifying costs, savings, and consequences for health and well-being, it could be examined whether investments in universal parenting interventions are a wise way of utilizing resources compared to other health initiatives.

The program in this trial, All Children in Focus, later referred to as the ABC-program as well (in Swedish - Alla Barn i Centrum), consists of structured, universal health-promotion group meetings that target parents of children aged 3–12 years. The purpose of the program is to strengthen the relationship between parents and their children. The ABC was developed from 2009 to 2011, and has been piloted during its development in twelve municipalities and city districts in the County of Stockholm, Sweden.

### Aims

The main objective of our study is to evaluate the effects of a universal parenting program, the ABC-program, through promotion of parental self-efficacy as well as children’s development and well-being. The following research questions are investigated:

1) What effects does participation in the ABC-program have on parental skills, self-efficacy, and children’s health immediately after, and 6 months post baseline?

2) Do program fidelity and delivery by group leaders mediate the outcome of the program?

3) Are either parental level of attendance at sessions or use of program components related to effects of the program immediately, 6, and 12 months post baseline?

4) Does impact of the program differ depending on the child’s gender, age, parental ethnicity, education, income, or mental health?

5) Does parental improvement in emotion regulation or self-efficacy mediate change in children’s health and development?

6) Is the ABC-program cost-effective?

7) Is self-rated parental competence related to observed parent and child behaviors?

8) Are there changes in the interaction between parent and child?

## Methods/Design

**Figure 1:**
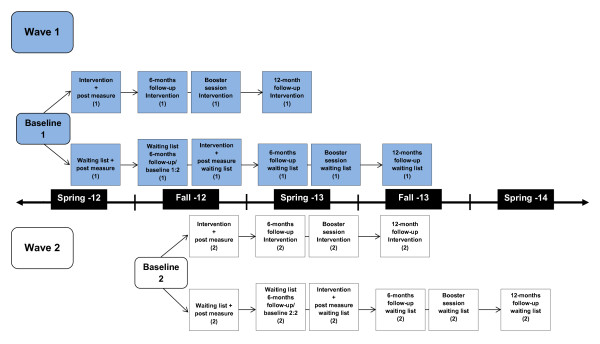
Timeline detailing the research procedure for the ABC-RCT.

The ABC-program is evaluated in a prospective, multicenter, randomized wait-list controlled trial (RCT), in a ratio of 1:1, to be allocated to the intervention group or a waiting-list control group. The RCT was undertaken after a first-time pilot study from 2009–2011 where 405 parents were recruited, after which the ABC-program and questionnaires included were slightly modified. Parents with children 3–12 years old in the municipalities and city districts taking part in the RCT-study will be asked to participate further, from 2012–2014. Those who accept participation after receiving verbal information and providing written informed consent, will be randomized to receive the ABC-program either directly or after six months (on a waiting-list). Parents randomized to a delayed-onset ABC-program starting after 6 months compose the control group. There will be approximately 300 parents each in the intervention and control group, respectively. Follow-ups are employed 6 and 12 months after baseline (See Figure 1).

### Setting

Parents with children ages 3–12 years were recruited in eleven municipalities and city districts in the County of Stockholm, Sweden. The participating municipalities and city districts represent a geographical coverage of different parts of the county, including different groups of parents regarding education, income, and ethnicity. The ABC-program is performed with trained personnel/ABC-group leaders at local agencies, such as preschools, schools, and family health centers.

### Participants, recruitment and randomization

Information about the study has been distributed through advertisements in local papers, supermarkets, child health centers, family centers, preschools, schools, as well as the websites of the participating municipalities and city districts. Joint materials such as posters and leaflets were available for use with recruitment efforts. The most common recruitment strategies were through personal contact with parents, information at schools and preschools, websites, advertisements, and a specially produced ABC promotion video. The video was shown in connection with the checkouts at grocery stores. Other strategies entailed letters to parents and contacts with maternity health services and child health services. Some local differences occurred; that is, a few of municipalities/city districts recruited in a more limited way, while most others recruited more comprehensively. Interested parents were invited to local information meetings at local premises such as schools and municipal buildings in February-March 2012 (Wave 1), and September-October 2012 (Wave 2). Meetings were held in the evenings, and parents were offered some beverages and snacks. The researchers have given information about the RCT-study, and group leaders or contact persons have explained about the content of the ABC-program at the information meetings. Parents were also informed about participation being voluntary, and that they could withdraw at any time without having to state a reason. Parents were also given printed information together with a consent form to be signed if they agreed to participate in the trial. The signed consent forms were collected, and afterwards baseline measures (via questionnaires) were taken. For parents not attending the meeting, information, consent, and baseline measure questionnaires were sent home with a pre-stamped envelope to be returned to the researchers. In Wave 1, 311 parents joined the study and in Wave 2, 310 parents joined.

**Figure 2:**
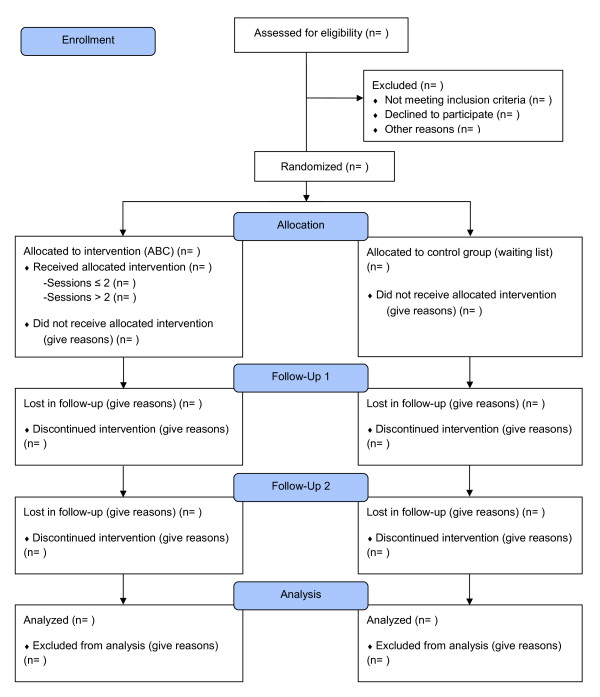
The ABC-RCT CONSORT flow diagram.

Randomization to allocate parents to intervention or control groups was performed after collection of baseline data (See Figure 2). In families where both parents announced interest to participate, both were invited to participate, but data will be pooled if their answers in the questionnaires cover the same child. Randomization was conducted at the level of individual parents for each municipality/city district: a 1:1 ratio, using SPSS version 20. However, couples were randomized together as one unit.

Parents participating in the trial received incentives during and after the trial. After completion of the first follow-up questionnaire, parents received entrance for the whole family to an open-air museum located in the city of Stockholm. About the same time, parents in the control group received a chocolate bar (parents in the first wave) and reflectors (parents in the second wave). When the final follow-up questionnaire has been conducted, 12 months after the baseline, parents will receive a gift card offering them either a paperback book or three movie rentals.

#### Recruitment of contact persons

Initially, one contact person was recruited from each municipality and city district to coordinate and to serve as a link between research and municipalities/city districts. Their tasks were to recruit group leaders and parents to the trial, as well as, to provide premises for information meetings and for ABC-groups.

#### Recruitment and training of group leaders

Most of the group leaders were recruited from each municipality/city district. In total 67 group leaders were recruited. Twenty seven of them received training during the pilot study of ABC, while 40 were trained during the RCT. By profession they were, for example, preschool teachers, social workers, and pedagogues.

During the trial, trainings were held for new group leaders, and led by ABC instructors; the other group leaders were trained by the developers (the method group described below) of ABC during the pilot study. Group-leader training lasted four and a half days, followed by continuous follow-up tutoring.

All group leaders were informed about the trial; new group leaders at their first training session and old group leaders were invited to meetings held by the researchers. Group leaders gave their written consent to participation in the trial, confirming that they were informed about the four tasks that they were obligated to participate in:

1. Video recording of one of the four sessions (this session was randomized by the researchers).

2. Filling in a group-leader checklist after each session. (The checklist comprised questions regarding how the session was performed).

3. Filling in an attendance list for parents participating in the sessions.

4. Delivering questionnaires to parents regarding their commitment to the method at the beginning of sessions two, three, and four.

#### Basic skills for group leaders

Each group is run by two group leaders, important skills for group leaders during the ABC sessions are to encourage and empower the parents, activate the discussion in the group, and structure the discussion to the topic. The skills are further illustrated in Table 1.

**Table 1:** Description of required basic skills for group leader

**Group leader skill**	**Purpose**	**Examples and methods**
**Validate**	Strengthen parents’ confidence in their own ability.	**Encourage** parents’ work with the ABC: "It sounds like you had a breakthrough moment with ABCD although the time was short, wonderful!"
	Be a model for the parents.	**Treat parents emphatically**: "It sounds like you’ve had a rough week, no wonder you feel tired."
	Create a permissive atmosphere so that parents dare raise difficulties.	**Encourage** parents’ responses and contributions.
**Activate**	Increase participation.	**Return questions**:
	Maintain the interest of the parents.	1. What are your thoughts about this?
		2. Does anybody else have any comments?
	Encourage activity of the parents when this is desirable.	Wait for the parents’ response. Give them time to think of discussion questions.
	Ensure that everyone gets the same opportunity to talk	Let the parents discuss in small groups.
**Structure**	Increase clarity.	**Start and end** the meeting on time.
	Stick to the content of the material and bring the discussion back to the theme.	**Write the agenda** on the board or flip chart.
		Go through the hits according to **group leader material**.
		**Cancel discussions that drift from the subject** and return to contents by thanking for the comments, summarizing and moving on.
		Talk in **behavioral terms**: “What does Karin *do* when she is angry?”, “What do you *do*?”

#### Group leader skills and fidelity

In order to study facilitator skills and fidelity to the ABC-program for group leaders, each pair of group leaders is video recorded. Before starting the group, randomization is made of which session of the total four is going to be recorded (with parental approval). Only the two group leaders are recorded visually on the video, while only the voices of participating parents are recorded; parental approval of the recordings has to be collected.

#### Drop out before intervention

During the first recruitment wave, 37 parents came to information meetings or got information sent home about the trial, but chose not to participate. On the second wave, there were 54 parents who did not want to participate in the trial after having received information. The most common reasons for not wanting to participate were time constraints, language barriers, and parents being indisposed to dates when the ABC-groups were to be held. A few parents were not interested in participating in research, while some parents did not state a reason for not wanting to participate. Very few parents declined participation because of the randomization procedure, which had made it uncertain whether they would be assigned to the same ABC-group as a friend, or because they wanted some other kind of support (e.g., individual support). Some parents also brought information home to their partners or to friends who they thought might also be interested in participating in the trial. A majority of these were interested and chose to participate. It should be noted that child care concerns were a barrier for some partner participation.

### Power and sample size

The intention has been to include 300 families in the intervention and control groups respectively. According to a sample size calculation, 107 parents are needed in each group to allow a detection of an effect size of 0.4 at 90% power and the p < 0.05 level of significance, with a one-tailed test. It is also of importance to take into account the effects of a cluster design, as the intervention is offered to groups while effects of this have to be identified in intra-class correlations (ICC) [23]. The design effects of this in a universal promotion parenting program are not identified earlier, while an estimate of 0.01 could be considered as conservative. This would require a sample of 220 parents in each group [23].

Families that do not fulfill participation in the ABC will also be followed together with families attending the intervention. An attrition rate of at least 20% is expected at the 6-month follow-up after baseline, in accordance with results from somewhat similar studies [24,25]; this is why an over-recruitment will be done.

### Intervention

ABC targets one of the most important protective factors for children – the parent–child relationship. The theoretical base is social learning theory [26], with focus on how children learn from their parents and how parent behavior can promote a positive relationship. Moreover, attachment theory [27] is included in relation to child-directed play to enhance parental attunement to the child. Influence of the external environment on family functioning has been incorporated by addressing parental stress and circumstances outside the family [28,29]. ABC consists of components included in parental programs demonstrated to be effective [11,30]. Also considered, were unpublished interviews with parents about content and factors that could contribute to participation in a program, as well as interviews with potential group-leaders. Moreover, a pilot study was performed with parents in order to test the feasibility of the components.

In this study, these components are being tested with a universal approach. ABC consists of four 2.5 hour structured sessions with approximately 10 parents in each group. The sessions are held every other week with homework between the sessions. After the six-month follow-up, a booster session is offered to the parents. The themes of the four sessions are: Showing love, Being there, Showing the way, and Pick your battles. In the first session, the parents set goals that are followed-up at the last session. Short films and role plays are used to facilitate discussions in the group. Each parent receives a copy of a binder with the content of the sessions with room to take notes. In the following section, the content of the four sessions are summarized.

### Session 1. Showing love

#### Goals

The main aim of the meetings is to promote children’s development in a positive direction. As long as children live at home, the relationship with their parents is the most important factor in their development. Positive family relationships provide protection for children when exposed to stressful or harmful experiences. *My goals*: It can be helpful to look ahead to see whether what we are doing now is consistent with what kind of parents we want to be.

#### Parental factors

Children are constantly learning new things. Two ways of explaining how children learn from their parents are the *role model factor* and the *attention factor*. *The role model factor* – Your child does what you do. For example, children will copy your words, tone of voice, how you act in various situations, body language, and fears. *The attention factor* – Your child will do more of what gets your attention. Children need love and attention to develop. They quickly learn what they should do to get their parents’ attention. If children are not given enough positive attention, there is a risk they will try to get negative attention instead.

#### Showing love

Children need to feel loved. Feeling loved gives children better self-esteem and protects them in times of difficulty. Love and warmth also strengthens relationships and reduces conflict within the family unit.

#### Five to one

It is important that there is more positive attention than negative attention given for a relationship to work. Five times more love is a good balance in all relationships. If there is a great deal of conflict in the relationship, the problem might be that the child needs more positive attention.

#### Focus on what works

Recognizing and paying attention to what works leads to *virtuous cycles* and less conflict. The child will seek less negative attention and there will be more opportunities for encouragement.

### Session 2. Being there

Spending time with your child will improve the relationship and reduce conflict. *Child in charge* – *ABCD* (*see below*): Time spent with the child when the parent allows the child to lead the activity is good for the child’s development. *ABCD* also promotes better cooperation between children and parents.

**A**ctivities lead by the child

**B**oost and encourage

**C**onnect with and describe child’s actions

**D**aily activity

#### The interaction chain

The interaction chain (Table 2) is a tool to help parents understand why children behave the way they do and how adults and children influence each other’s behavior. Parents can also use the interaction chain to see how they can help the child by doing things differently *before* and *after*.

**Table 2:** Description of the interaction chain at session 2

**Before**	**After**
*Preparations*	*Attention*
Prepare your child for what is going to happen.	Increase attention and encouragement when it works!
Choose an appropriate time for the activity.	
*Participation*	
Reach a joint agreement with your child on what tasks they will have.	
Give your child time to perform their tasks.	
*Positive encouragement*	
Tell your child what they should do, not what they should stop doing. For example: ‘Come sit here beside me’ instead of ‘Stop running around’.	
*Routines*	
Establish routines – do things the same way every time.	
*Positive expectations*	
Show that you believe your child is capable!	

### Session 3. Showing the way

By keeping calm during times of conflict, we model and show the way to our children, although it is not always so easy to do. All parents get angry at their children once in a while, which is normal. It is, however, important to try to handle the anger in a way that will not worsen the situation or causes more conflicts in the future.

#### Annoyance and anger

The drawbacks of shouting include: *The role model factor* - the child learns to shout; *The attention factor* - the child receives a great deal of attention when there is an argument, leading to vicious cycles and more conflicts. Further drawbacks include: *The interaction effect* - the relationship deteriorates and the family atmosphere worsens,; *The* “*cry wolf*” *effect* - sharp reprimands lose their effect, which can be dangerous in situations when your child must listen immediately to avoid danger; *The spiral effect* - an angry reprimand often triggers an angry retort.

#### Showing the way

It can be hard to always be good role models for our children, even though we do not want to get angry. There are often several things that affect how we act. A few examples:

General stress - When we are under stress, we get angry more easily. *Things you can do*: Change what can be changed. Lower your standards, or accept what cannot be changed. Make sure to schedule time for yourself for recovery and exercise.

Critical situations - Critical situations are those which lead to anger more often than others. *Things you can do*: Think about your own critical situations so that you can prepare for them.

Thoughts and physical reactions - Anger affects our bodies and how we think. It becomes more difficult to think clearly and find solutions to problems. *Things you can do*: Learn to recognize your own early signs of anger, since they are then easier to manage.

Behavior - There are drawbacks to expressing your anger in action. *Things you can do*: Take a break, anger is an emotion that will dissipate by itself if you simply wait a while. Talk about what made you angry later. Find solutions together with your child.

Consequences - What feels right and works for the moment is not always good over the long term: the short-term trap.

### Session 4. Pick your battles

When parents work together and think about which battles are important and which you can choose not to engage in, it gets easier to be consistent and things become clearer to the child. Paying more attention to what works while cutting down on nagging and reprimands will eventually lead to less conflict.

#### Natural consequences

Sometimes, as an effect of choosing not to engage in battles, your child will have to take the natural consequences instead. For instance, children often decide to wear their gloves when they realize that their hands get cold without them.

*Things will get worse before they get better*. If a parent stops nagging or reprimanding, the child may at first react more strongly in an attempt to get a reaction. If the parent can still refrain from nagging or reprimanding, the conflicts will subside.

Validate, explain, and distract - Some battles are too important to choose not to engage in. Validate, explain, and distract is a description of how you can meet your child in a manner that reduces the risk of conflict with the child.

**Validate your child’s feelings**. Show that you understand, and put the child’s feelings into words.

**Explain and repeat why**. Give your child a brief explanation for what he/she should consider.

**Distract**. Give your child something else to do and encourage that instead.

### Measures

Time points of primary interest for all outcome measures are at baseline, two weeks after intervention, and six months after baseline for both intervention and control groups. In the intervention group only, a twelve month follow-up will be conducted. A minor group of parents and children will be observed, to explore any changes in the dyadic interaction between parent and child after taking part in the ABC-program. Video recordings of both a mealtime and a play situation will be collected at baseline and at six months after baseline.

In the survey questionnaire, parents provide information about their gender, marital status, number of children, birth country, educational level, monthly income, child’s age, and gender, birth country for the child, and child care. The questionnaires included in the survey are described below.

#### Primary outcome measures

*Parental Self*-*Efficacy* (PSE) measure parents’ perception of their parenting on a 48-item questionnaire rated on an 11-point Likert scale, ranging from 0 = completely disagree to 10 = completely agree. The questionnaire is composed of 8 subscales adapted from a Tool to Measure Parenting Self-efficacy – TOPSE, which encompasses positive emotion, being with your child, empathy, guiding, rules, pressures, acceptance, and experience [31]. The questionnaire was translated into Swedish and then back-translated by an authorized translator to make sure that the original content was kept in the translation. A first draft of the questionnaire including 82 statements was tested in a pilot group of parents not participating in ABC (n = 11) who were interviewed about the content and ease of response. All parents had remarks about most of the negatively formulated items, and some of the items were considered to be unclear. The feedback from the parents was taken into account in a revised questionnaire with 34 statements removed (unpublished data).

The revised questionnaire with 48 items was tested in the pilot study of ABC including parents with children ages 1–14 years (n = 405), and analyses of internal consistency were performed. The alpha coefficients were .91 for the whole scale, and varied from .66 for the subscale pressures to .83 for the subscale being with your child.

*Child Health and Development*. To measure parent reports of children’s health and development, a questionnaire influenced by KIDSCREEN with 35 items and 6 dimensions rated on a 5-point scale to measure a child’s physical and mental health, emotional development, independence, family relations, and social competence was developed. The questions assess frequency of behavior/feelings (never-seldom-sometimes-often-always) and intensity of attitudes (not at all-slightly-moderately-very-extremely) [32]. The questions about child health and development were tested in a pilot study (n = 405). Analyses of internal reliability were computed with alpha coefficients of .92 for the total scale and subscales ranging from .73 for physical health to .87 for mental health.

#### Secondary outcome measures

*Program fidelity and delivery skills* by group leaders are measured by observations of filmed program sessions. The group leaders are rated according to two dimensions. One is regarding competence in terms of framework, speaking with own terms, being well-prepared, presenting the program in a positive manner, activating parents, strengthening parents, and interaction between the two group leaders. The second dimensions cover the content of the program including presentation of the theme, presentation of goals, dimensions of parenting, discussion of the theme, and testing at home. Group leaders are also filling in a checklist after each session containing questions on program fidelity and parental engagement.

*Parent’s use* of the method is measured through number of attended group meetings in questionnaires two weeks after each group meeting by questions about the use of program components (“yes/no”), and how it worked (“Positive/Both positive and negative/Negative”).

*Parenting Practices Interview* (PPI) [33,34] assesses retention of family rules as well as positive and negative parenting practices. In this study, the two subscales parental praise incentives, and harsh parenting were used; they were covered by 26 items, rated on a 7-point scale (from “never” to “always”, and from “not likely at all” to “extremely likely”, depending on the wording of the question). The two subscales from the PPI were tested during the development of the ABC program with a group of parents (n = 142). Analyses of internal reliability revealed that the alpha levels were .75 for parental praise and .78 for harsh parenting.

*The General Health Questionnaire*-GHQ12 [35] is included to measure the parents’ mental health, scored on a 4-point Likert scale (Positive items - “better/more than usual”, “same as usual”, “less than usual” and “much less than usual”; Negative items – “not at all” no more than usual”, rather more than usual” and “much more than usual”). The alpha level in the pilot study with 405 parents was .91.

*The Dyadic Adjustment Scale* (DAS). The brief version of the DAS covered by the subscale Dyadic Satisfaction is used to measure relational satisfaction/dissatisfaction between parents [36]. Four items on a 7-point scale, ranging from “never” to “always”, and “extremely unhappy” to “extremely happy” (dependent on the wording of the question) is used. Internal validity for the scale was .82 in the pilot study including 299 parents.

*The Emotion Regulation Questionnaire* (ERQ) evaluates the parental emotion regulation strategies [37]. This is an established and validated 10-item self-report questionnaire rated on a 7-point Likert scale (1 = strongly disagree, 4 = neutral, and 7 = strongly agree). Individuals are asked to rate the extent to which they typically try to think or behave differently in situations in order to regulate their emotions. Alpha levels were .81 for the subscale reappraisal and .74 for the subscale suppression in the pilot study with 292 parents.

A *Visual Analogue Scale* (*VAS*) is used for the health economic evaluation [38]. The health variable which will be explored to evaluate cost-effectiveness is health-related quality of life (HRQOL) in children measured by a parent-proxy VAS-scale rated from 0 to 100.

#### Observations of parent–child interaction

Video recordings of a mealtime and a play situation of the parent and child together are conducted in the home of the families. In total, 19 parents and their children are included in the observations. Concerning the mealtime, the families are asked to record a dinner, and for the play situation the families are borrowing a drawing board (Etch A Sketch) from the researchers. Parents receive instructions with the drawing board on how to use it together with the child. At both the mealtime and the play situation, the child is asked to be recorded from the front, and the parent from the front or in profile. Families who have their own video camera are encouraged to use that, or otherwise a camera is borrowed from the researchers. Observations are conducted twice: before parents participate in ABC, and about six months after first being observed.

### Analysis

Analyses will be conducted both with intention-to-treat-analyses as well as study-completer-analyses. The statistical analyses will be performed according to best practice guidelines for evaluating effects in RCTs [39]. This may come to include MANOVA, repeated measures ANOVA, and regression analyses. Cohen’s *d* will be included to calculate effect sizes. The importance of some of the parental characteristics will also be evaluated in relation to the 6-month-outcome – in other words, the moderator as well as the mediation relations. To do this, the definition by Baron and Kenny [40] for a mediational relation will be used: Evaluate 1) whether there is a significant correlation between the independent and dependent variables, 2) if the independent variable and possible mediator are significantly related, and 3) if the mediator and dependent variable are significantly related. Since the waitlist group was offered the intervention after six months, pre- to post- data for intervention and waitlist groups will also be combined to improve power in the secondary analyses.

Data collected in the trial will also be used to evaluate the possible cost-effectiveness of ABC. The cost-effectiveness analysis (CEA) [41] will include costs, savings, and health gains of the children. Calculations will be performed from the societal perspective meaning that the intention is to consider all costs and consequences. The foundation of the CEA will be an incremental analysis to investigate the extra costs, potentially extra savings and health gains of ABC in relation to the control group. The costs will include resources needed for running the ABC-program such as project staff salaries, parents’ time, materials, and meeting arrangements. The time spent on learning and performing the ABC-program will be based on information from contact persons at the local agencies involved in the study. The health gains will be reported in gained Quality Adjusted Life Years (QALYs), which will be estimated by potentially-improved scores on the HRQOL among children pre-intervention, immediately post-intervention, and 6- and 12 months post-intervention. A sensitivity analysis will be performed to explore the uncertainty of the study result.

The video-recorded observations of parents and children will be analyzed applying two different approaches. First, to explore if there are any changes in the dyadic interaction between parent and child after taking part in the ABC-program. The second approach will be to compare parental reports of parental self-efficacy as well as child health and development with observed behaviors.

### Ethics

This study obtained ethical approval from the Regional Ethics Committee at the Karolinska Institutet (Registration number: 2012/93-31/5). The same committee gave ethical approval to the amendment, including video-recorded observations of parents and children (Registration number: 2012/2184-32).

### Working groups

#### Research group

A research group including an associate professor in clinical psychology, two doctors of medicine, and two PhD students was established to conduct the trial. The competences of the group comprised knowledge of child mental development, public health, parenting programs, health promotion, health economics and experience conducting and evaluating randomized controlled trials.

#### Method group

Responsible for the delivery and implementation of the method and training of group leaders, was a group of three registered psychologists within family and social welfare in the City of Stockholm. The group also had the main responsibility for the development of the ABC-program, although in consultation with the research group in the early stages. This approach was based on earlier research that has demonstrated larger effects when evaluations are performed by implementers of an intervention [42,43].

#### Steering group

Beyond parts of the research and method groups, the steering group also consisted of representatives from two of the participating municipalities/city districts. The aim of the group was to monitor the process of the trial.

#### Reference group

In the reference group, members of the research group participated, as well as ABC developers and the contact person for each municipality/city district. Additionally, a representative from the County Administrative Board in the County of Stockholm was included. A main task for the reference group was to disseminate information to all involved municipalities/city districts.

### Adjustments

In the first recruitment wave, the baseline measure consisted of a paper questionnaire. For the second wave, a web-based questionnaire was introduced as a complement. This resulted in about one-third of the baseline measures for the second wave being conducted online. Most of these web-based questionnaires were completed by parents who could not attend the information meeting.

## Discussion

This paper describes the protocol for a randomized controlled trial evaluating the effectiveness of a Swedish universal parenting group program, ABC (All Children in Focus/Alla Barn i Centrum (Swed.)). All Children in Focus aims at improving children’s health and development, and includes four 2.5 hour structured sessions for parents with children aged 3–12 years old. Except for the quantitative outcome measures to evaluate the effectiveness of ABC, the protocol also describes health economic and qualitative analyses of the program. The inclusion of several methodologies in the evaluation of ABC contributes to a deepened knowledge and understanding of potential use of ABC in the future, as well as for future evaluations of universal health-promotion parenting programs.

The design of this study is a randomized controlled trial with a waitlist control group. This type of evaluation can raise concern over some practical considerations. Especially in the case of working with other professionals in addition to researchers, the practitioners in our study were concerned about parents entering the control group, and having to wait six months to get the intervention. It is important for all involved in the study to understand the ethics and the reasoning for this. As described from earlier pragmatic trials on child mental health interventions conducted in Wales, there are some factors which can contribute to participation by service settings, such as early involvement, and clarification of requirements and contributions of both researchers and the service settings [44]. In the trial of ABC, most practitioners were involved at an early stage since they participated in the pilot studies of the intervention; only three of the participating municipalities and city districts were newly introduced to the randomized trial when it started. Before the trial started, all contact persons were informed about what the municipality/city district had to contribute to the trial (e.g., group leaders, premises, recruitment of parents), and all group leaders were informed about the trial. In this manner, participating municipalities/city districts were involved, and clarification could be made where it was needed to prevent future confusion about expectations.

The training and tutoring of group leaders was performed either by two ABC instructors, or by the method group. It is crucial that all group leaders are trained and tutored in a similar way, as was done for this trial. To measure fidelity to the program, video recordings of the group leaders will be used, performed by themselves (in a few cases one of the researchers supplied the group leaders with the equipment). The group leaders had to sign a consent form that they were willing to participate in recording of one session. Initially, some of the group leaders expressed concern about the recordings, but they were convinced about the advantages by other group leaders who had already tried this in the pilot study. So far, two pairs of group leaders have not video-recorded any session, which has been due to a mistake in communication, and problems with the recording equipment. Also, for some pairs of group leaders, the whole sessions were not recorded due to trouble with the equipment; in addition, a few group leaders recorded another session than the one randomized for the group.

When recruiting parents, all involved in a project must be aware of what the research trial implicates and what it means for those participating. As an example, some parents expressed that they wanted to participate together with a friend or a neighbor. Parents should be informed about the study procedure at an early stage to avoid confusion; for example, not all participants will receive the intervention directly, but may be assigned to a waiting-list control. In this trial, there was confusion about the study procedure for some parents.

To increase the possibility of a high response rate in the study, incentives were used in the form of free family entrance to an open-air museum after the first follow-up, and also a chocolate bar or reflectors for the waiting-list subjects. For the last follow-up, a gift certificate for either a paperback book or movie rental is distributed. Less than half of the families took advantage of the entrance to the open-air museum when this was offered; it is uncertain to what extent these incentives may have influenced the response rate.

Some of the outcome measures included in this study, such as GHQ or PPI, have mainly been used to assess reduction of problems or symptoms rather than the increase of strengths or positive behaviors. In health promotion research more instruments measuring positive aspects of health would be worthwhile.

Most of the existing parenting programs [7-12] tend to focus on treatment or prevention of different child behavior problems. Other parenting programs, which are offered at a universal level [16,17,25], seem to have a more pathogenic approach, meaning that they focus on prevention of ill-health or problems rather than having a salutogenic perspective with focus on promotion of health. This study will thereby add knowledge in the field of health promotion regarding the potential value of a universal health promotion parent group program.

## Competing interests

The authors declare that they have no competing interests.

## Authors’ contributions

LL conceived and designed the study as well as drafted and revised the manuscript. MU participated in the design of the study and coordination of data collection as well as assisted in drafting and revision of the manuscript. CJ and ES helped to design the study and drafted the manuscript. ML and KLR designed the intervention, and contributed to the method section of the manuscript. AM participated in the design of the study, critically revised the manuscript and will perform the economic analyses. PE conceived and designed the study and helped to draft and to revise the manuscript. All authors have read and approved the final manuscript.

## Pre-publication history

The pre-publication history for this paper can be accessed here:

http://www.biomedcentral.com/1471-2458/13/688/prepub
